# Serious Infection Following Anti–Tumor Necrosis Factor α Therapy in Patients With Rheumatoid Arthritis: Lessons From Interpreting Data From Observational Studies

**DOI:** 10.1002/art.22808

**Published:** 2007-09

**Authors:** W G Dixon, D P M Symmons, M Lunt, K D Watson, K L Hyrich, A J Silman, British Society for Rheumatology Biologics Register Control Centre Consortium

## Abstract

**Objective:**

In a recent observational study, we found that the risk of serious infection following anti–tumor necrosis factor α (anti-TNFα) therapy in patients with rheumatoid arthritis (RA) was not importantly increased compared with the background risk in routinely treated RA patients with similar disease severity. Observational data sets are, however, subject to a number of important biases related to selection factors for the timing of starting and stopping therapy. Infection risk is also likely to vary with duration of therapy. This study was undertaken to examine the influences of these biases and of the method of analysis on the risk of infection.

**Methods:**

We compared the risk of serious infection in 8,659 patients treated with anti-TNFα with that in 2,170 patients treated with traditional disease-modifying antirheumatic drugs (DMARDs) recruited to the British Society for Rheumatology Biologics Register. We applied a number of statistical models in which we varied the length of the followup period by using different definitions of the date of discontinuation of treatment and different lag periods of risk following drug cessation.

**Results:**

When the at-risk period was defined as “receiving treatment”, the adjusted incidence rate ratio comparing patients receiving anti-TNFα therapy with patients receiving DMARD therapy was 1.22 (95% confidence interval [95% CI] 0.88–1.69). Limiting followup to the first 90 days, however, revealed an adjusted incidence rate ratio of 4.6 (95% CI 1.8–11.9). Rates of infection were increased in the 90 days immediately following drug discontinuation and beyond, explained by selection factors for drug discontinuation.

**Conclusion:**

These findings show that overall, the way in which UK rheumatologists select patients for starting and discontinuing anti-TNFα therapy explains our previous finding of no increase in risk. However, there may be important increases in true risk, notably early in the course of treatment, that would become more evident depending on the definition of at-risk period.

There are currently 3 anti–tumor necrosis factor α (anti-TNFα) drugs licensed for use in rheumatoid arthritis (RA) in the UK: infliximab and adalimumab, both monoclonal antibodies, and etanercept, a TNFα receptor fusion protein. Since TNFα is involved in host defense and tumor surveillance, there have been concerns that anti-TNFα therapy might lead to adverse events, particularly infection and malignancy. These are complex issues, since RA itself increases the risk of serious infection and certain malignancies, acting either via the disease process or secondary to traditional disease-modifying antirheumatic drugs (DMARDs). The question that needs to be addressed is whether anti-TNFα therapy further increases that risk.

The long-term safety of treatment with biologic response modifiers cannot, however, be addressed in short-term randomized clinical trials, not only because such trials have a limited duration, but also because they recruit insufficient numbers of patients to detect rare events. Large population-based registers are thus increasingly being used to study drug safety (1–4).

A number of methodologic aspects of register study design are now well-established. There must be a comparison cohort of patients who are as similar as possible to patients in the treatment cohort, aside from taking the drug in question. Adverse events must be reported in a robust manner with avoidance of reporting bias. Information on potential confounders should be collected and adjusted for in the analysis. There are, however, obvious selection factors in determining which patients start, and indeed stop, a particular therapy, which are not necessarily captured even in intensive data sets. Residual confounding is a major concern. In 2 recently published studies from Germany and the UK (2,5), the infection rates observed in the anti-TNFα cohorts were very similar, but the 2 groups of investigators drew very different conclusions, the former estimating a doubling of risk and the latter no increased risk. These conclusions highlight important differences between the 2 countries in their methods of selecting comparison cohorts.

There are also a number of issues relating to the method of analysis that are often ignored, but which need to be considered when interpreting the data from individual registers. This study examined the influence of different approaches on the analysis of serious infection rates following anti-TNFα therapy in patients with RA.

The first key question is whether any increase in risk of infection is constant over time, or whether there are specific times when the risk is higher or lower. Plausible models of risk over time include increased risk of an adverse event on initial exposure, constant risk with ongoing drug exposure, or increasing risk with cumulative exposure to the drug (Figures 1a–d). The pattern of risk is likely to differ according to the adverse event considered. An infusion reaction may be more likely to occur early in the course of therapy, whereas a malignancy may be related to cumulative drug exposure. For any given adverse event, the overall risk pattern may be a composite of these patterns.

**Figure 1:**
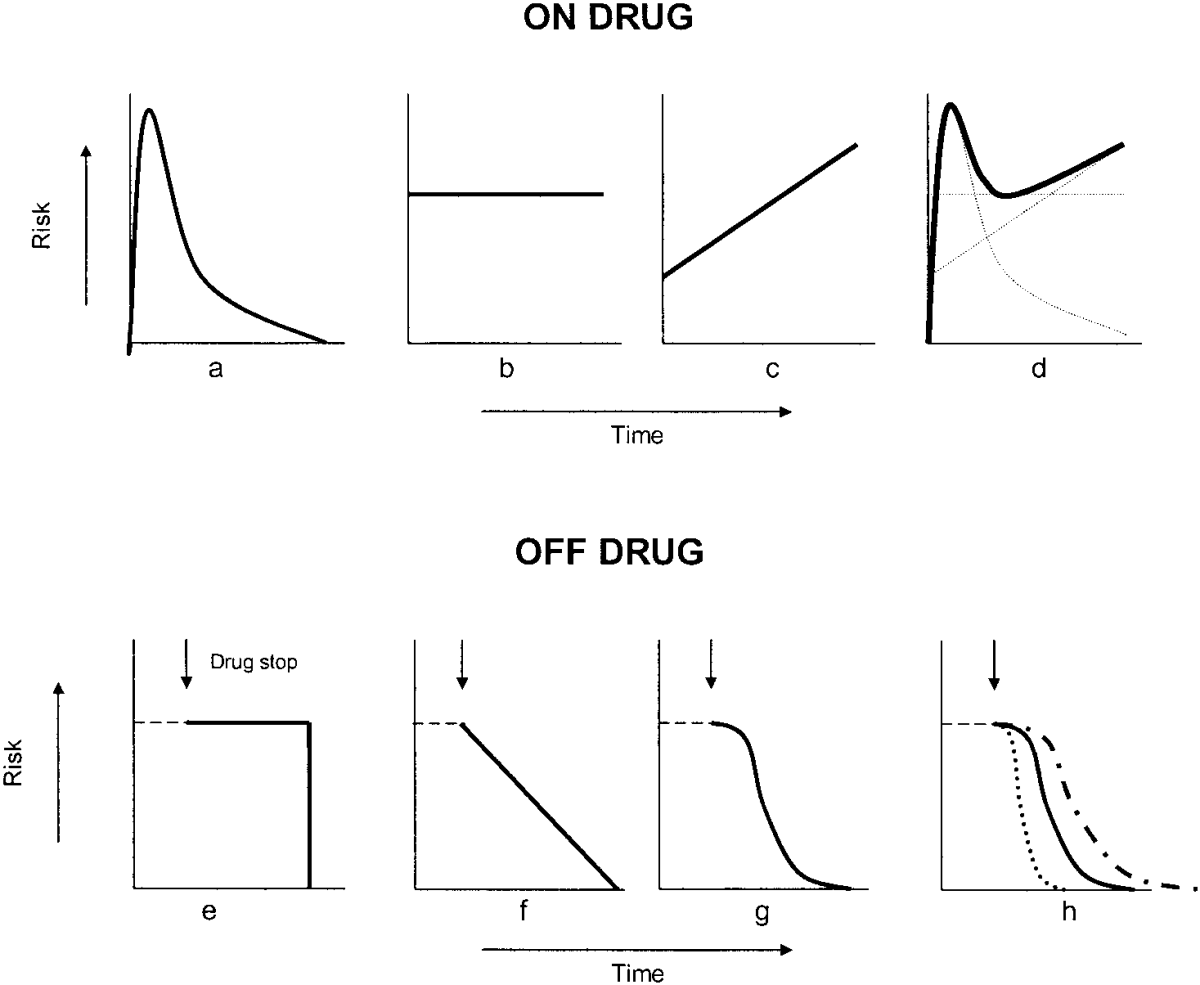
Patterns of constancy of risk of infection while receiving treatment (on drug) and after discontinuation of treatment (off drug). **a**, Increased risk at start of therapy. **b**, Constant risk with ongoing drug exposure. **c**, Increasing risk with cumulative drug exposure. **d**, Combination of the risk patterns shown in **a**–**c**. **e**, Ongoing constant risk for set lag window after discontinuation of treatment (drug stop). **f**, Linear decrease in risk back to baseline. **g**, Nonlinear decrease in risk back to baseline. **h**, Differing durations of risk windows, based on the half-life of each drug.

It is also necessary to define an at-risk window, that is, the period when adverse events should be attributed to a drug. The minimum plausible at-risk window would extend from the beginning of therapy to the therapy discontinuation date (Figure 2a). Defining this, however, is complex in the context of anti-TNFα therapy, given the administration schedule (with, in the case of infliximab, for example, infusions several weeks apart). In addition, depending on the pharmacokinetics and pharmacodynamics of the drug, should the at-risk window extend beyond the drug discontinuation date (Figure 2b)? The third concern is whether a drug confers a long-term risk beyond its period of pharmacologic activity. In this case, an “ever taken drug” model would be applicable (Figure 2c). In combination, these factors can have substantial influences on the measured risk.

**Figure 2:**
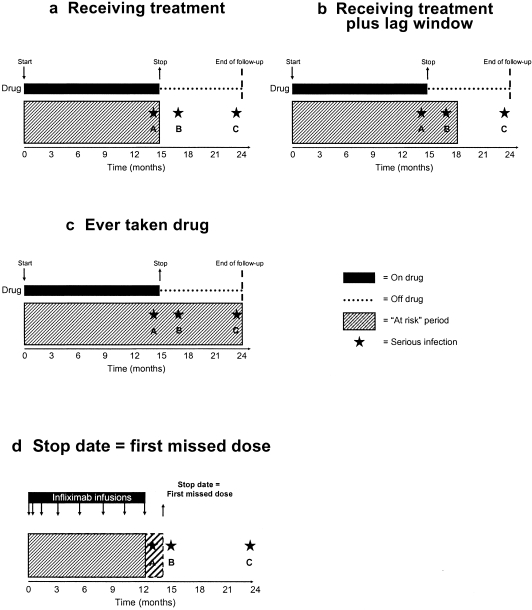
Influence of the definition of the at-risk period on an infection being attributed to therapy. **a**, Duration of treatment as the at-risk period. The at-risk window during which events can be attributed to the drug runs from 0 to 15 months. Serious infection A (at 14 months) is therefore attributed to the drug, but serious infections B (at 17 months) and C (at 23 months) are not. **b**, Duration of treatment plus a lag window of 3 months beyond discontinuation of the drug as the at-risk period. Serious infections A and B are attributed to the drug, but serious infection C is not. **c**, Start of treatment to the end of followup as the at-risk period. Serious infections A, B, and C are all attributed to the drug. **d**, Date of the first missed dose as the drug discontinuation date (stop date). If the stop date is incorrectly defined as the date of last dose given, rather than the first dose missed, event A will not be attributed to the drug.

Further, the risk once the patient has stopped taking the drug may take one of many patterns (Figures 1e–h). It may remain constant, may decrease back to baseline linearly or, more likely, decrease in a nonlinear manner. It is possible that risk may never return to the pretreatment baseline level.

Finally, the statistical approach to analysis of changing risk pattern over time also needs to be considered. In the analysis of rare events, data may need to be aggregated, or “smoothed,” to obtain a meaningful estimate of risk over time. Interpretation of risk over time may be affected by this smoothing process.

Without carefully considering these sources of variability, the simple description of infection risk as x cases per 1,000 person-years of therapy is impossible to interpret. We used a large national observational study to assess the impact of the various issues addressed above on the estimated risk of infection following anti-TNFα therapy in patients with RA.

## PATIENTS AND METHODS

### The British Society for Rheumatology Biologics Register (BSRBR)

The BSRBR is a national prospective observational study that was established with the primary aim of examining the medium- to long-term safety of biologic response modifiers used in the rheumatic diseases. The methodology has been described in detail elsewhere (5,6). Briefly, it consists of a cohort of patients with RA treated with anti-TNFα, and a comparison cohort of patients with active RA who receive traditional DMARD therapy and have never taken biologic response modifiers. The latter cohort exists in the UK because of budgetary constraints on the prescription of anti-TNFα drugs (7). Members of the BSRBR Control Centre Consortium are shown in Appendix A.

Data on demographic characteristics, disease severity and duration, drug therapy, and comorbidity are collected at baseline from both cohorts. All patients are then followed up using 3 parallel methods. First, consultants are sent a questionnaire every 6 months, requesting details of all changes in therapy and all adverse events that have occurred in that period. Second, patients are sent a 6-month diary in which to document all hospital admissions, new medications, and new hospital referrals. Third, all patients are flagged with the UK General Register Office, which provides the BSRBR with information on deaths. Serious infections, reported from any of these 3 sources, are defined as those that led to death or hospitalization or required intravenous antibiotics.

To be included in the present study, patients had to have been followed up for ≥6 months prior to July 31, 2006. Followup time was censored at the last completed followup prior to July 31, 2006, or the date of switching to a second biologic response modifier (or first biologic response modifier for the comparison cohort), or death, whichever came soonest. In other words, patients who switched to a second or subsequent anti-TNFα drug contributed time and adverse events data to their first drug only.

### Statistical analysis

Baseline characteristics of the anti-TNFα cohort were compared with those of the DMARD cohort, using Wilcoxon's rank sum tests for continuous variables and chi-square tests for categorical outcomes. Between-drug comparisons were made using Kruskal-Wallis rank tests for continuous variables. The incidence of serious infection was then compared both between the anti-TNFα cohort and the comparison cohort and between individual anti-TNFα agents. Rates of serious infection per 1,000 person-years were calculated using a large series of assumptions relating to the issues described above. In each instance, incidence rate ratios were calculated using Poisson regression, comparing the anti-TNFα cohort with the DMARD cohort. All analysis was conducted using Stata, version 8.2 software (StataCorp, College Station, TX).

## RESULTS

### Baseline characteristics

There were 10,755 patients included in the analysis (8,659 treated with anti-TNFα and 2,170 treated with DMARDs only). Seventy-four patients switched from the DMARD cohort to the anti-TNFα cohort and were included in both groups. The baseline characteristics of the 2 cohorts are shown in Table 1. The DMARD cohort included proportionally more men, and patients in this cohort were older and, as expected, had less severe disease.

**TABLE 1:** Baseline characteristics of the DMARD-treated and anti-TNFα–treated patients*

Characteristic	DMARD (n = 2,170)	All anti-TNFα (n = 8,659)	Etanercept (n = 3,844)	Infliximab (n = 2,944)	Adalimumab (n = 1,871)	*P*†
Age, mean ± SD years	60 ± 12	56 ± 12‡	56 ± 12	56 ± 12	57 ± 12	0.052
Sex, % female	72	76‡	77	76	74	0.035
DAS28 score, mean ± SD	5.0 ± 1.4	6.6 ± 1.0‡	6.6 ± 1.0	6.6 ± 1.0	6.5 ± 1.0	0.006
HAQ score, mean ± SD	1.5 ± 0.8	2.1 ± 0.6‡	2.1 ± 0.6	2.1 ± 0.5	2.0 ± 0.6	<0.001
Disease duration, median (IQR) years	7 (1–15)	12 (6–19)‡	12 (6–19)	12 (6–19)	11 (5–19)	0.009
Extraarticular RA, no. (%)	415 (19.1)	2,541 (29.3)‡	1,123 (29.2)	896 (30.4)	522 (27.9)	0.131
Baseline steroid use, no. (%)	418 (19.3)	3,793 (43.8)‡	1,784 (46.4)	1,342 (45.6)	663 (35.4)	0.016
Diabetes, no. (%)	132 (6.1)	470 (5.4)	230 (6.0)	134 (4.6)	106 (5.7)	0.031
COPD/asthma, no. (%)	416 (19.2)	1,130 (13.1)‡	536 (13.9)	361 (12.3)	233 (12.5)	0.082
Smoking history, no. (%)						
Current smoker	537 (25)	1,886 (22)§	797 (21)	650 (22)	436 (23)	0.045¶
Former smoker	849 (39)	3,298 (38)	1,454 (38)	1,107 (38)	733 (39)	
Never smoked	767 (35)	3,431 (40)	1,568 (41)	1,169 (40)	690 (37)	

* Seventy-four patients switched from the cohort treated with disease-modifying antirheumatic drugs (DMARDs) to the cohort treated with anti–tumor necrosis factor α (anti-TNFα) drugs and were counted in both groups. Smoking history was available for 2,153 patients treated with DMARDs and 8,615 patients treated with anti-TNFα drugs. DAS28 = Disease Activity Score in 28 joints; HAQ = Health Assessment Questionnaire; IQR = interquartile range; RA = rheumatoid arthritis; COPD = chronic obstructive pulmonary disease.

† Across 3 anti-TNFα drugs.

‡ *P* < 0.001 versus DMARD-treated patients.

§ *P* for trend < 0.001.

¶ *P* for trend.

### Risk of infection while receiving therapy

There were 1,089 serious infections in total: 114 in the comparison cohort and 975 in the anti-TNFα cohort, 737 occurring while the patients were receiving therapy. Using the “receiving treatment” model of analysis (Figure 2a), the crude rate of serious infection was 39.2 per 1,000 person-years in the DMARD cohort and 55.5 per 1,000 person-years in the anti-TNFα cohort, ranging from 50.4 to 63.0 events per 1,000 person-years in the 3 anti-TNFα drug cohorts (Table 2). For this “receiving treatment” analysis, the at-risk period extended from the start date of anti-TNFα treatment to the first missed dose.

**TABLE 2:** Rates of serious infections obtained using models that include different definitions of the treatment-attributable at-risk period*

	DMARD (n = 2,170)	Etanercept (n = 3,844)	Infliximab (n = 2,944)	Adalimumab (n = 1,871)	All anti-TNFα (n = 8,659)
Model A, receiving treatment					
Person-years	2,908	6,021	5,034	2,221	13,277
No. of infections	114	308	317	112	737
Rate per 1,000 person-years (95% CI)	39.2 (32.3–47.1)	51.2 (45.6–57.2)	63.0 (56.2–70.3)	50.4 (41.5–60.7)	55.5 (51.7–59.5)
Adjusted incidence rate ratio (95% CI)†	Referent	1.15 (0.82–1.61)	1.28 (0.91–1.81)	1.17 (0.81–1.69)	1.22 (0.88–1.69)
Model B, duration of treatment plus 90-day lag window					
Person-years	2,908	6,274	5,226	2,323	13,823
No. of infections	114	361	354	127	842
Rate per 1,000 person-years (95% CI)	39.2 (32.3–47.1)	57.5 (51.8–63.8)	67.7 (60.9–75.2)	54.7 (45.6–65.0)	60.9 (57.0–65.0)
Adjusted incidence rate ratio (95% CI)†	Referent	1.26 (0.91–1.74)	1.35 (0.97–1.89)	1.24 (0.87–1.78)	1.30 (0.93–1.78)
Model C, ever received treatment					
Person-years	2,908	6,998	5,874	2,548	15,420
No. of infections	114	432	405	138	975
Rate per 1,000 person-years (95% CI)	39.2 (32.3–47.1)	61.7 (56.0–67.8)	68.9 (62.4–76.0)	54.2 (45.5–64.0)	63.2 (59.4–67.2)
Adjusted incidence rate ratio (95% CI)†	Referent	1.34 (0.97–1.86)	1.41 (1.02–1.97)	1.25 (0.88–1.77)	1.35 (0.99–1.85)

* 95% CI = 95% confidence interval (see Table 1 for other definitions).

† Adjusted for age, sex, disease duration and severity, extraarticular rheumatoid arthritis, baseline steroid use, diabetes, chronic obstructive pulmonary disease, and smoking history.

After adjustment for age, sex, disease duration and severity, extraarticular RA, baseline steroid use, diabetes, chronic obstructive pulmonary disease, and smoking history, there was no significant difference in risk of infection between any of the anti-TNFα cohorts and the comparison cohort. These results are consistent with the findings published in our previous report on serious infection rates, with followup to September 2005 (5). In this observational study, the treating rheumatologist was aware of the patient's therapy at the time of infection, possibly leading to a lower threshold for admission into the anti-TNFα cohort. However, such a bias does not explain these findings.

### Constancy of risk

**Figure 3:**
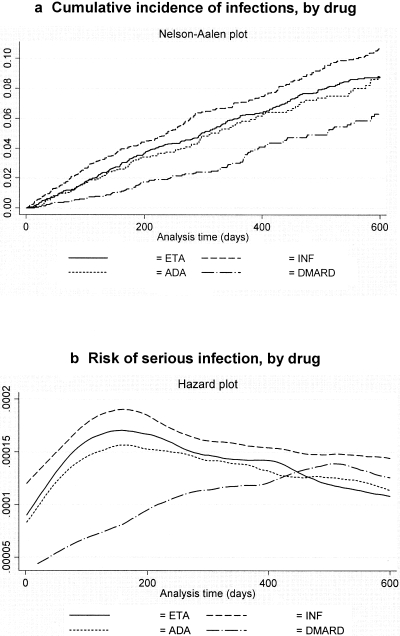
Cumulative incidence of infections (**a**) and risk of serious infection (**b**), by drug. The y-axis shows the hazard, or risk, of serious infection. ETA = etanercept; INF = infliximab; ADA = adalimumab; DMARD = disease-modifying antirheumatic drug.

A plot of the cumulative incidence of serious infections while receiving treatment (Figure 3a) showed that at any given point in time, it was more likely that patients in the anti-TNFα cohorts had a serious infection compared with patients in the comparison cohort. This does not necessarily mean that the absolute risk was higher at all time points in the anti-TNFα cohorts. In order to explore this, and to evaluate the change in risk over time (as in Figure 1), a plot of the slope of the curves in Figure 3a, or hazard plot, was constructed. This showed a marked increase in hazard in the anti-TNFα cohorts, peaking at ∼6 months, but declining over time (Figure 3b).

To further explore a possible early risk of infection (Figure 1a), the rates of serious infections were analyzed with followup censored at 90 days after the start of anti-TNFα therapy (or 90 days after registration date in the DMARD cohort) (Table 3). The adjusted incidence rate ratios for the 3 anti-TNFα drugs compared with the DMARD cohort showed an ∼4-fold or greater risk of serious infection in the first 90 days. These ratios were much higher than the incidence rate ratios seen for the entire followup period (Table 2).

**TABLE 3:** Rates of serious infections obtained using model A: receiving treatment, limited to first 90 days of exposure*

	DMARD (n = 2,170)	Etanercept (n = 3,844)	Infliximab (n = 2,944)	Adalimumab (n = 1,871)	All anti-TNFα (n = 8,659)
Person-years	532	917	723	451	2,091
No. of infections	13	55	69	27	151
Rate per 1,000 person-years (95% CI)	24.4 (13.1–41.4)	60.0 (45.5–77.4)	95.4 (75.0–119.2)	59.9 (39.8–85.9)	72.2 (61.5–84.2)
Adjusted incidence rate ratio (95% CI)†	Referent	4.1 (1.5–10.8)	5.6 (2.1–15.1)	3.9 (1.3–11.2)	4.6 (1.8–11.9)

* 95% CI = 95% confidence interval (see Table 1 for other definitions).

† Adjusted for age, sex, disease duration and severity, extraarticular rheumatoid arthritis, baseline steroid use, diabetes, chronic obstructive pulmonary disease, and smoking history.

This result is biologically plausible, suggesting an increased early risk of infection in the anti-TNFα cohort. However, as shown in Figure 3b, the increased incidence rate ratios in the first 90 days appeared to be driven not only by the elevated crude rates of infection in the anti-TNFα cohorts, but also by the low early crude rates in the DMARD cohort. Both cohorts, but particularly the anti-TNFα group, were screened before initiation of treatment, to avoid inclusion of patients who had an imminent serious infection. However, such a difference between the groups in prescreening would bias against an increased risk in the anti-TNFα group and does not explain this early increased risk.

Selection factors also apply after recruitment. If a patient in the anti-TNFα cohort developed an incident serious infection, the physician made an active treatment decision about whether therapy should be continued once the acute infection had resolved. Some patients were considered to be at high risk of a future infection and did not resume therapy. Therefore, the anti-TNFα cohort in a “receiving treatment” analysis had a progressively lower risk of infection over time because of the selective exclusion of high-risk patients. This may explain the gradual reduction in risk from the 6-month time point onward.

Because of clinical decisions influencing the observed pattern of risk, it is very difficult to ascertain the true pattern of risk, and a possible real increase in cumulative risk may be hidden by the selection factors described above. Thus, over the period of observation, the decisions made in clinical practice in conjunction with the underlying serious infection risk of the drugs did not lead to an increasing risk of serious infections over time. This is not the same as concluding that these agents do not lead to an increase in infection risk. Exploration of the fluctuation of serious infection risk in between doses of the drugs is made virtually impossible by these same factors.

### Influence of choice of date of discontinuation of treatment

Within the BSRBR, stop dates for all 3 anti-TNFα drugs are based on clinical records. An accurate definition of the stop date is particularly important for infliximab, given the prolonged interval between infusions. In ∼90% of records, the given stop date for infliximab was the last infusion date. However, an adverse event caused by the drug (apart from an infusion reaction) is unlikely to occur at the time of an infusion, and thus the stop date should be the first missed dose, rather than the last dose given. Indeed, such events are often the reason for discontinuing the drug. If the date of the last dose given were used as the stop date, events which followed the last infusion (event A in Figure 2d) would not be attributable to the drug in the analysis, grossly underestimating the event rate. Defining the stop date as the last dose given for infliximab leads to an infection incidence rate of 49.0 per 1,000 person-years, much lower than the 63.0 per 1,000 person-years shown above. Thus, for all BSRBR analyses, including the “receiving treatment” analysis (Table 2), the stop date was defined as the first missed dose.

### Influence of lag window

Once treatment is discontinued, the risk associated with the drug may not immediately return to the predrug baseline. To address this, the at-risk window was extended for an arbitrary period beyond the first missed dose. Thus, in the following analysis, events occurring in the 90 days after the stop date (first missed dose) for each of the 3 drugs were considered attributable to that drug, as in Figure 2b.

The crude rates of serious infection in this analysis ranged from 54.7 to 67.7 per 1,000 person-years for the 3 anti-TNFα drugs (Table 2). These rates were higher than those found when the analysis was restricted to the “receiving treatment” period for all 3 drugs (Table 2). In fact, the rate of serious infection was higher in the 90-day window after drug discontinuation than during the treatment period. The rates per 1,000 person-years (95% confidence intervals [95% CIs]) in the 3 cohorts in this 90-day window after discontinuation of anti-TNFα therapy were 202 (153–256) for etanercept, 193 (139–256) for infliximab, and 147 (85–231) for adalimumab. The incidence rate ratios (95% CIs) in the first 90 days after discontinuation of treatment compared with the rates while receiving treatment were 3.3 (2.4–4.5) for etanercept, 2.8 (1.9–4.1) for infliximab, and 2.5 (1.3–4.8) for adalimumab.

These findings are counterintuitive, since the risk of serious infection would be expected to decrease once anti-TNFα treatment was discontinued. This could be explained by a rebound in disease activity after discontinuation of the drugs. However, the major reason for the increased risk in this 90-day window became clear on reviewing the individual case histories: the infection was causally related to the reason for discontinuing the drug. In the majority of cases, the reason was an adverse event. The subsequent infection may have been either a direct or an indirect consequence of the initial adverse event, e.g., an aspiration pneumonia following a stroke, or an opportunistic infection following chemotherapy for a malignancy. In other cases (for example, prior to surgery), discontinuation of treatment was planned. This event is associated with its own inherent increased risk of infection, elevating the risk in the time period after discontinuation. Further, the drug may have been discontinued because of symptoms of a serious infection, though the infection was only diagnosed some time later. This last scenario would also have resulted in an underestimation of the rate of serious infections in the anti-TNFα cohort in a “receiving treatment” analysis, and the analysis of the 90-day lag window might be a better reflection of the true rate.

The influence of these selection variables on serious infection during the lag window makes it very difficult to explore the true pattern of infection risk for the anti-TNFα drugs once discontinued (Figures 1e–h). Such considerations also make it hard to compare the differing patterns between the drugs. For example, is there a longer lag window of risk for infliximab compared with etanercept, given its longer half-life? While it is possible to calculate the rates for lag windows set differently for the 3 drugs based on multiples of their half-lives, these rates are very difficult to interpret given the presence of other factors, such as the reason for drug discontinuation.

### Continued risk after discontinuation of treatment

An important question to address in relation to anti-TNFα drugs is, assuming there is an increased risk of certain adverse events while taking the drug (and during a lag phase after discontinuation), whether that risk returns to baseline or is persistent. If it is persistent, for how long does the risk continue? Such an ongoing risk would be particularly relevant for adverse events such as malignancy, though less so for serious infections. It would be expected that any effect that anti-TNFα drugs have on susceptibility to infection would subside within months of discontinuing the drug. One way to explore this is to look at the serious infection rate for the entire followup period, regardless of the date of drug discontinuation (Figure 2c). Using this model, the rates of serious infection for the 3 anti-TNFα drugs were 61.7 per 1,000 person-years for etanercept, 68.9 per 1,000 person-years for infliximab, and 54.2 per 1,000 person-years for adalimumab (Table 2). These rates were also higher than those found using either the “receiving treatment” or the “duration of treatment plus 90-day lag window” analyses, as discussed above.

Again, these results seem counterintuitive. As with the lag phase analysis, the reason for drug discontinuation may influence the rate of subsequent infection, but this influence should decline with time. For example, if the treatment is stopped for planned surgery, the postoperative infection risk declines with time, and infection risk should return to presurgical levels.

The reason for the increased risk in this model may relate to the active treatment decisions made in an observational study. For patients who have a serious infection while receiving anti-TNFα therapy, clinicians will decide whether treatment should be resumed, based on their opinion of further infection risk. Thus, there is a “healthy drug continuers” or “depletion of susceptibles” effect, and restricting analysis to those who continue the drug selectively retains those at the lowest risk.

## DISCUSSION

In a recently published report (5) we showed that the overall risk of severe infection was not increased following anti-TNFα therapy. In this extended analysis it is clear that there are several influences that make it very difficult to generate a robust answer to this superficially simple question. The risk of infection attributable to anti-TNFα therapy measured in an observational study cannot be adequately summarized as a single estimate. Large national registers have the capacity to reveal some of the patterns of adverse events that might otherwise be hidden behind such a point estimate.

It is important to be specific about aspects such as time period of interest during therapy, the choice of stop date, what allowance should be made for continuing pharmacologic action, and how to evaluate risk following these periods. Although standardization of analytical approach will help in comparing the findings of different studies, selection factors for both starting, and as we have shown, discontinuing therapy may seriously compromise interpretation.

In the future, defining stop dates will become even more difficult. Rituximab, an anti-CD20 B cell–depleting drug, is now licensed for the treatment of RA (8). It is given in 2 infusions 2 weeks apart, with its effects lasting for ∼6–12 months. When can a patient be considered to be receiving or not receiving this treatment?

Despite these concerns, use of a consistent methodologic approach in all population registers is essential. However, it is equally important to avoid making oversimplistic conclusions about the infection risk conferred by these drugs.

## AUTHOR CONTRIBUTIONS

Dr. Symmons had full access to all of the data in the study and takes responsibility for the integrity of the data and the accuracy of the data analysis.

**Study design.** Dixon, Symmons, Watson, Hyrich, Silman.

**Acquisition of data.** Dixon, Symmons, Watson, Hyrich, Silman.

**Analysis and interpretation of data.** Dixon, Symmons, Lunt, Silman.

**Manuscript preparation.** Dixon, Symmons, Lunt, Hyrich, Silman.

**Statistical analysis.** Dixon, Lunt, Silman.

## ROLE OF THE STUDY SPONSOR

The BSRBR was established primarily to investigate the safety of biologic agents in routine practice. The financial support to the BSRBR comes indirectly from the following UK companies marketing biologic agents in the UK: Schering-Plough, Wyeth Laboratories, Abbott Laboratories, and Amgen, but the independence of the BSRBR and its investigators is assured in the following manner. The resources used to fund the BSRBR are received under contract by the BSR, which then provides a research grant under a separate contract to the University of Manchester, allowing the investigators normal academic freedom in relation to the data, their analysis, and use. Under the terms of the contract between the BSR and the sponsoring pharmaceutical companies, all publications are sent in advance to the companies prior to submission, for the purposes of information. The companies can, if they wish, point out factual errors. Any comments are vetted by 3 members of the steering committee, who decide whether they should be passed on to the authors. All publications are also reviewed by the BSR, but the material presented and the views expressed in all publications from the BSRBR are those of the authors and do not necessarily represent the views of the BSR.

## APPENDIX A

### BSRBR CONTROL CENTRE CONSORTIUM

The BSRBR Control Centre Consortium consists of the following institutions (all in the UK): Antrim Area Hospital, Antrim (Nicola Maiden); Cannock Chase Hospital, Cannock Chase (Tom Price); Christchurch Hospital, Christchurch (Neil Hopkinson); Derbyshire Royal Infirmary, Derby (Sheila O'Reilly); Dewsbury and District Hospital, Dewsbury (Lesley Hordon); Freeman Hospital, Newcastle-upon-Tyne (Ian Griffiths); Gartnavel General Hospital, Glasgow (Duncan Porter); Glasgow Royal Infirmary, Glasgow (Hilary Capell); Haywood Hospital, Stoke-on-Trent (Andy Hassell); Hope Hospital, Salford (Romela Benitha); King's College Hospital, London (Ernest Choy); Kings Mill Centre, Sutton-In-Ashfield (David Walsh); Leeds General Infirmary, Leeds (Paul Emery); Macclesfield District General Hospital, Macclesfield (Susan Knight); Manchester Royal Infirmary, Manchester (Ian Bruce); Musgrave Park Hospital, Belfast (Allister Taggart); Norfolk and Norwich University Hospital, Norwich (David Scott); Poole General Hospital, Poole (Paul Thompson); Queen Alexandra Hospital, Portsmouth (Fiona McCrae); Royal Glamorgan Hospital, Glamorgan (Rhian Goodfellow); Russells Hall Hospital, Dudley (George Kitas); Selly Oak Hospital, Selly Oak (Ronald Jubb); St. Helens Hospital, St. Helens (Rikki Abernethy); and Withington Hospital, Manchester (Paul Sanders).
